# Thermal Behavior of an Asphalt Pavement in the Laboratory and in the Parking Lot

**DOI:** 10.1155/2015/540934

**Published:** 2015-03-12

**Authors:** J. B. Martinkauppi, A. Mäkiranta, J. Kiijärvi, E. Hiltunen

**Affiliations:** University of Vaasa, 65200 Vaasa, Finland

## Abstract

The urban, constructed areas are full of buildings and different kinds of pavements and have a noticeable lack of trees and flora. These areas are accumulating the heat from the Sun, people, vehicles, and constructions. One interesting heat collector is the asphalt pavement. How does the heat transfer to different layers under the pavement or does it? What are the temperatures under the pavement in Finland where the winter can be pretty hard? How can those temperatures be measured accurately? These are the main questions this paper gives the preliminary answers to. First the thermal behavior of asphalt and the layers beneath are researched in the laboratory and then the measurement field is bored and dug in the parking in the Western coast of Finland, 63°5′45′′ N. Distributed temperature sensing method was found to be a good choice for temperature measurements. Thermal behavior of pavement has been monitored in different layers and the preliminary results have been published here. The goal of this research is to assess the applicability of asphalt pavements for heat energy collection.

## 1. Introduction

Urban energy means accumulation of energy already existing in urban, built, and constructed areas. One of its approaches is asphalt energy in which the heat energy is collected under asphalt layer. Asphalt heat energy collection is an exciting approach since asphalt pavements cover large areas in cities and roads thus making a huge reservoir already available for use. Different aspects of asphalt and heat have been already studied in many publications and even patents have been issued: the first patent is already from 1979 [1]. One of the aspects is the effect of heat on the asphalt structure like cracking [2] or healing [3] or thermal behavior [4]. Other aspects are the possible use of asphalt as latent heat storage [5] or as a source of heat energy.

Asphalt solar collectors have been suggested not only to collect heat but also to prevent the so-called heat island effect and structural damage of pavement due to thermal cycles with temperature as high as 70°C [6]. The heat island effect depicts the phenomenon where the asphalt heats up the air above causing an increase in the need of air conditioning and a decline in air quality [6, 7]. An asphalt solar collector is made by installing pipes inside the asphalt. Fluid circulation inside the pipes causes heat to be transferred from warmer pavement to cooler fluid thus lowering the temperature of the pavement. Heat is extracted from the low temperature fluid using the heat pump. Solar heating systems are effective especially in buildings with low energy demand for heating [8]. In the development of the new constructions with good insulation and low energy demand the use of heat pump system is a good alternative [9]. Typical applications of asphalt collector systems are snow-melting systems (see, e.g., [10]) and thermal uses in nearby buildings. The underfloor heating system with heat pump is the most economic and ecologic solution [11] and is widely used in the low energy systems.

A laboratory set-up with sand, gravel, and asphalt layers was made to recognize the heat transfer through the layers and possible heat accumulation inside some layers. The purpose of the set-up is to provide some understanding of behavior of different materials under heating as well as to be a preliminary test for pavement temperature measurements [12]. The heating was applied in four occasions on the set-up while measuring the temperature of different layers. Since the results were promising, a temperature measurement system has been constructed on the pavement in the parking lot with five wells of different depths. Reference wells are located on the lawn field. In the Nordic countries the winter and, particularly, the soil freezing are the issues which have to be taken into account while installing and utilizing the collector system under the pavement. The impacts of soil freezing are monitored in the pavement measurements in the parking lot in Vaasa, Finland, too.

In this paper, the selected temperature measurement method, laboratory set-up, and constructed pavement temperature measurement system are described in Section 2. Measurements are described in Section 3.  Section 4 provides an analysis of the laboratory and Section 5 analyses the pavement measurement data. Conclusions are drawn in Section 6.

## 2. Measurement Arrangements

### 2.1. Temperature Measurement

Distributed temperature sensing (DTS) was selected for temperature measurements. It uses optical fiber as a sensor element and this provides possibility to collect temperature data from the whole fiber cable. This is advantageous since several other temperature measurement methods provide only data values at a certain measurement point. DTS has been shown to be feasible for monitoring soil moisture on an experiment with a sand filled tub [13].

DTS is based on emitting short pulses of laser and then detecting backscattered light [14, 15]. Part of backscattered light depends on temperature and based on this, the temperature for points of fiber cable is calculated by the DTS device. The measurement device used was Oryx DTS device [16]. The manufacturer data sheet gives the accuracy of an Oryx DTS device to be ±0.5°C. The Oryx DTS device was configured for 1 m spatial resolution which indicates that the position of measurement point can be located within 1 m accuracy. The operation mode was set to two channel measurements, with 5-minute measurement time for each channel. These settings produce data with 6 measurements per hour.

The optical fiber used here had bare fiber diameter of 50 *μ*m, diameter of 125 *μ*m with cladding, and diameter of 250 *μ*m with acrylate primary coating. Its product details are multimode, Ultra-Fox Plus. To monitor the room temperature during measurements, a different sensor, a PT100 platinum resistance temperature sensor, accuracy ±0.25°C, was employed.

### 2.2. Laboratory Set-Up

**Figure 1:**
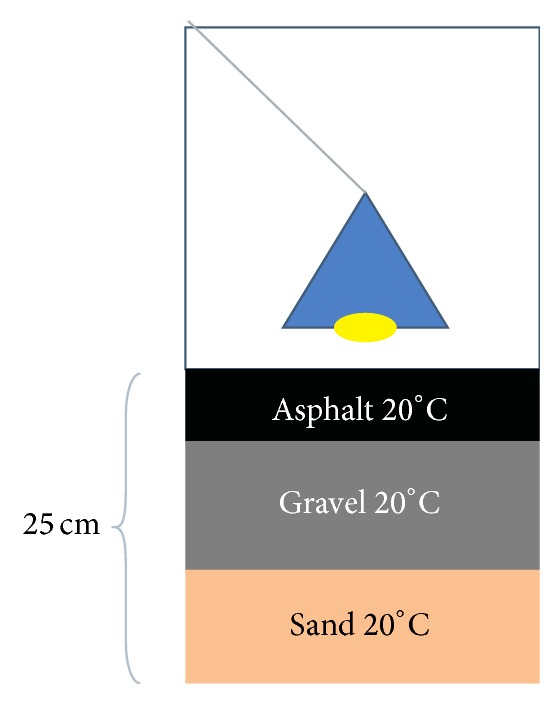
Experimental laboratory set-up for asphalt measurements.

The measurement set-up was built in Technobothnia Research Centre, which is a joint centre of the University of Vaasa, Vaasa University of Applied Sciences, and Novia University of Applied Sciences. The room temperature of the laboratory was kept around +20°C.  Figure 1 displays the experimental arrangement: under a 60 W heating lamp there were three layers of material: asphalt, gravel, and sand. Thickness of the asphalt layer was 5 cm while other layers were 10 cm thick. The set-up was made inside a plastic tub (size 80 liters).

**Figure 2:**
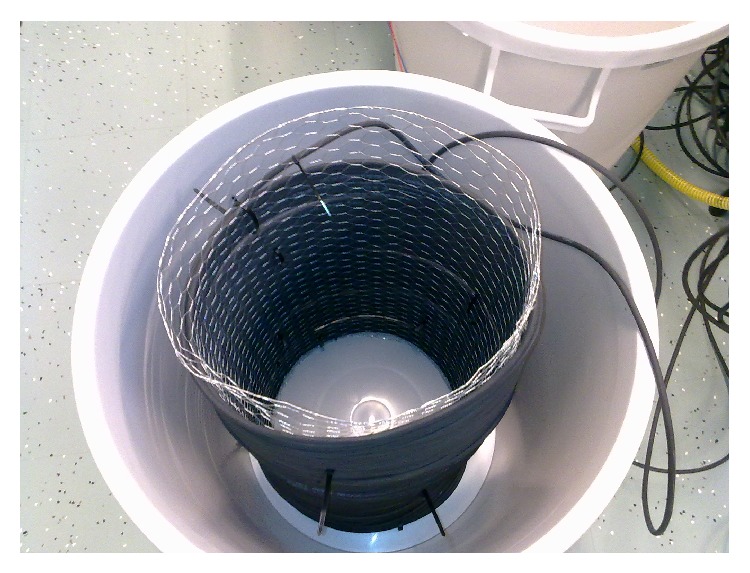
The cable was wrapped around a metal frame which was positioned inside the plastic tub.

The cable with optical fibers inside needed to be first centralized in the tub vertically. The optical fiber cable was thus wrapped around a round frame made from metal net (Figure 2) which was adjusted to middle of the plastic tub.

**Figure 3:**
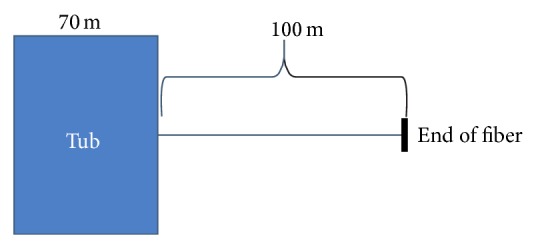
Schematic for cable arrangement.

The length of the cable is 525 m and it consists of two optical fibers joined at the end of the cable; thus the length of optical fiber is 1050 m. However, only 70 m of cable was possible to wrap around the metal net up to the height of 0.5 m. This means that wrapping 1.4 m cable produces a 1 cm rise. The simple schematic for cable is shown in Figure 3; note that parts of the cable were not inside the tub since the cable was not cut. In Table 1, the ranges of the cable are shown with corresponding material layer.

**Figure 4:**
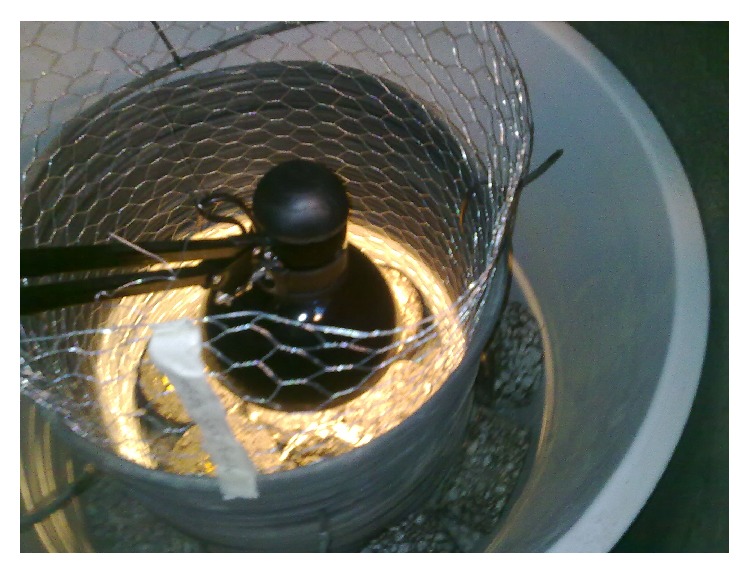
The set-up for measurements.

Cellular plastic (Nomalen 30, PE 30 kg/m³) was used as an insulation around the plastic tub. Thermal conductivity of Nomalen 30 is 0.038 W/(m·K) and thickness is 15 mm (NMC Cellfoam Oy 2013). The bottom was also insulated. After adding insulation, sand, gravel, and asphalt layers were added to the tub. The asphalt layer consisted of disc with diameter of 0.10 m and height of 0.05 m.  Figure 4 shows the final set-up which was used in measurements.

### 2.3. Pavement Set-Up

**Figure 5:**
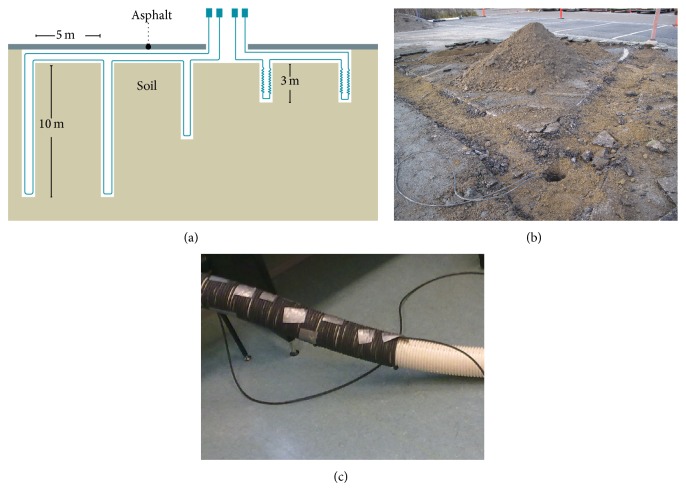
(a) This basic diagram indicates drilled holes in the asphalt pavement. (b) Installing cable to a 10 m hole. (c) The wrapping of cables for 3 m deep holes.

Five holes were drilled to the pavement; a diagram is shown in Figure 5(a). Depths of the holes are the following: two holes with 10 m depth, one hole with 5 m depth, and two holes with 3 m depth. The optical measurement cable was installed in each hole as displayed in Figure 5(b), and later the cables were connected together. For 3 m holes, the installed cable is wrapped around a pipe (Figure 5(c)). No wrappings were done for other holes. The aim was to drill holes and install the cables and cover the holes with soil. But the reality was that the walls of hole collapsed quite easily after the iron cover pipe for drilling was taken off. Because of this to enable the installation of cables the plastic cover pipes were used to keep the holes open. The plastic pipe was left to the hole. The cable was installed in the pipe and the pipe and the whole well were filled with soil. Only the hole of 5 m depth was installed without the plastic pipe. The 3 m depth holes remained open for the required time for installation and they were also implemented without cover pipe.

## 3. Measurements

### 3.1. Measurement at the Laboratory

The dry asphalt tub was subjected to measurement for a 4-day period. The asphalt layer was heated with a 60 W lamp bulb (see Figure 4). The duration of heating is shown in Table 2.

### 3.2. Measurement at the Pavement

**Figure 6:**
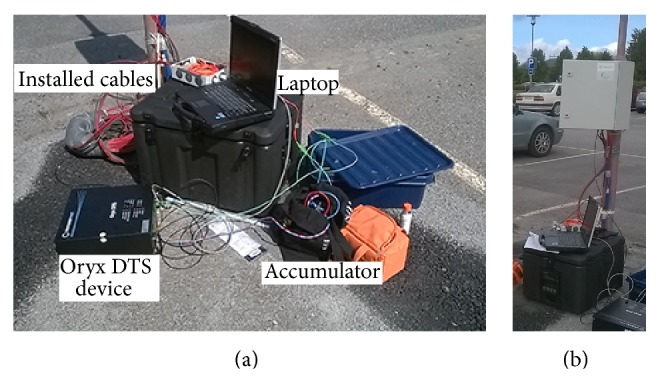
(a) Set-up for asphalt temperature measurement. (b) Measurement station.

The Oryx DTS device was used to collect the data from the installed cables. The measurement set-up is shown in Figure 6(a). Total length of installed cable was 553 meters including 3 splices. Two fibers were used in measurements. Accumulator was used as a power source. Measurements were carried out once per month in April and May. The data was acquired for a 10-minute period in 15-second sections per channel which means 20 measurements per fiber totally.

The connectors of fibers are located in the measurement station (Figure 6(b)). Later the data acquisition of heat flux plate will be installed too.

## 4. Analysis of the Laboratory Measurements

### 4.1. Period of Heating

**Figure 7:**
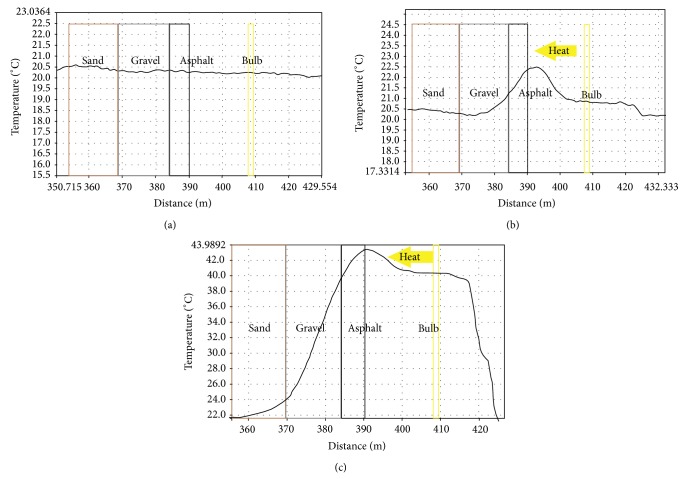
(a) The temperatures of sand, gravel, and asphalt layers are approximately at the room temperature before the heating starts (time stamp of data: 15.1.2013, 8:00; room temperature 20.06°C). (b) Four minutes after heating starts, warming of asphalt layer surface is clearly observed and thermal gradient is formed (time stamp of data: 15.1.2013, 8:20; room temperature 20.25°C). (c) The sand layer is also warmer after the heating period of 5 hours and 4 minutes (time stamp of data: 15.1.2013, 13:20; room temperature 20.57°C).

The temperature of material layers in the tub was measured before heating and the results are shown in Figure 7(a). Some temperature variations exist but they are relatively minor and all layers are approximately in the same temperature (±0.2°C).  Figure 7(b) displays the situation 4 minutes after starting the heating: the asphalt layer starts warming up and thermal gradient has formed across it. The heat is beginning to conduct to the lower layers. After the heating, the surface of asphalt layer has almost reached the temperature of +44°C as shown in Figure 7(c). Even the lowest layer, sand, is now slowly starting to warm up. The air gap between asphalt and lamp is also getting warmer partly due to heat energy reflected from the asphalt surface.

### 4.2. Cooling and Heating Cycles

**Figure 8:**
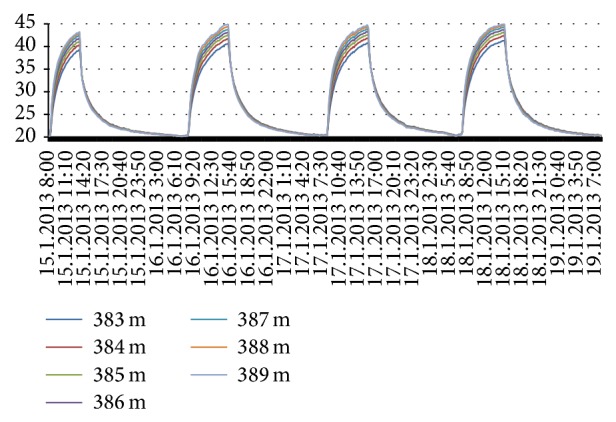
Heating-cooling cycles for asphalt.

**Figure 9:**
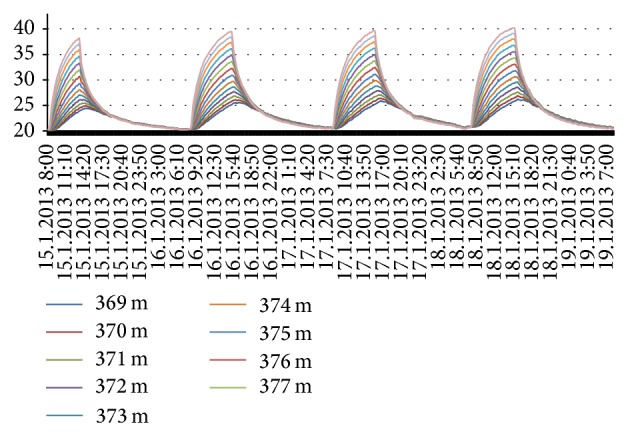
Heating-cooling cycles for gravel.

**Figure 10:**
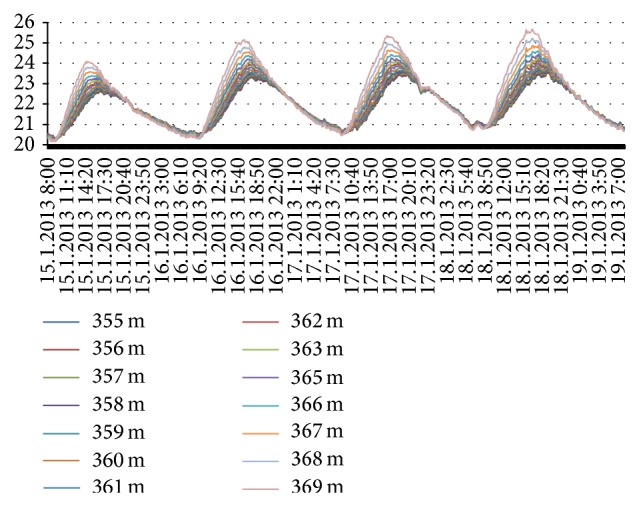
Heating-cooling cycles for sand.

The asphalt layer was subjected to heating in four occasions. After heating, the material layers were allowed to cool down towards the room temperature. The temperatures were measured with a 10-minute interval with DTS-method during these times. These heat-cooling cycles are displayed for asphalt layer in Figure 8, for gravel layer in Figure 9, and for sand layer in Figure 10. In each figure, the measurements have been made from several cable positions and the rounded values of cable positions from the beginning of the cable are indicated on the right side of the figures.

The thermal behavior seems to depend on position of the material at a layer during heating while being independent during cooling. The structure of material has naturally an effect: temperature variations in coarse gravel layer are larger than the two other layers. The asphalt layer as well as gravel layer reaches the prevailing room temperature during cooling time (Figures 8 and 9). However, the sand layer seems to store some of the heat energy as its temperature remains higher (Figure 10).

## 5. Analysis of the Pavement Measurements

**Figure 11:**
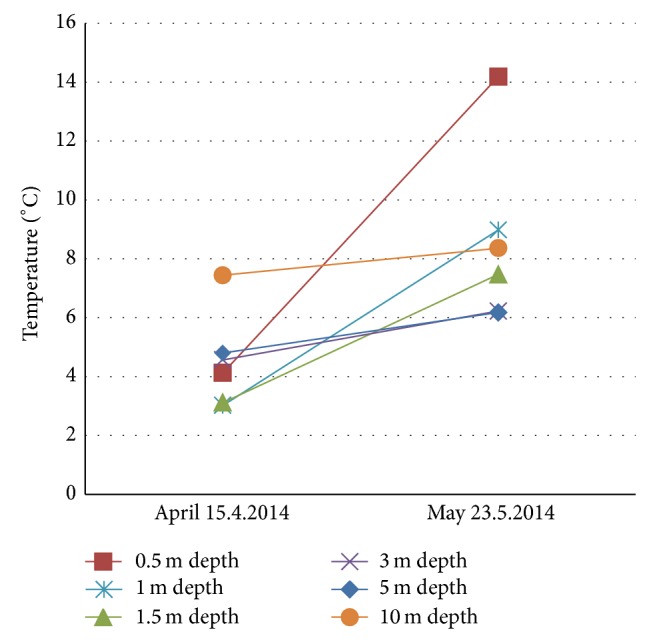
Temperatures under the pavement.

Temperatures of layers at different depths (0.5 m, 1.0 m, 1.5 m, 3 m, 5 m, and 10 m) were measured under the pavement in April and May 2014. The experimental field for asphalt heat energy research has been installed in spring 2013 and it will be later subjected to monitoring at every month. The goal here is to show that even one month will cause change in temperatures under different depths under asphalt. The measured values are shown in Figure 11. The temperature measurements in April still show marks of frost in the depths of 1–1.5 meters but the upper layer in the 0.5 m depth is affected by the outside temperature which according to the Finnish Meteorological Institute was 3-4°C on average in Vaasa region in April 2014.

In May the weather was warmer than a month before, the average outside temperature was 8–10°C [17], and warming up can be seen in the three uppermost layers (0.5 m; 1 m; 1.5 m) too. The end of May was again warmer than the beginning of the month and during daytime the temperature was higher than during night. The upmost layer is most affected by diurnal variation. The temperature rise at depths of 0.5 m, 1.0 m, and 1.5 m can be mainly attributed to the absorbed solar radiation of surface. Instead in the 3 m, 5 m, and 10 m depth only slight increasing of temperatures can be observed (Figure 11). The temperatures at depths of 3 m and 5 m are quite the same thus indicating a region where the heat conduction is more uniform and the temperature rise is only about 1°C.

The solar radiation and other seasonal factors affect the temperatures of ground approximately to the depth of 15 m according to the Geological Survey of Finland [18]. At the depth of 10 m, a large part of heat energy has geothermal origin and thus maintains its temperature at least 5°C. However, the location affects the prevailing minimum temperature. As can be seen in Figure 11, the temperature at the depth of 10 m has risen up to about 8°C.

## 6. Conclusions and Discussion

An interesting possibility for the heat energy collection is the asphalt pavements covering large areas in cities. The thermal behavior of asphalt pavement must be researched before the utilization of heat can be carried out. A laboratory set-up as well as real pavement DTS measurement system has been made as preliminary tests.

Heat conduction through three layers, asphalt, gravel, and sand, is studied first in a laboratory experiment. The layers were subjected to four heating-cooling cycles while measuring the temperatures via DTS-method. The slopes of heating and cooling curves (Figure 10) differ from each other clearly indicating dissimilar properties of different layers. The results indicate that while the upmost layer, asphalt, cools down relatively fast to the prevailing temperature, the lowest layer, sand, seems to store some of the heat energy. Thermal behavior for material inside a layer seemed to be dependent on the height level of the material related to the top of the layer during heating period while this was not observed during cooling period. The material content of a layer has naturally an effect on results. Coarse gravel had highest variation in its heat profile.

Since the results of laboratory experiments were very promising, a DTS measurement system was constructed on an asphalt pavement with 5 holes. Two-month preliminary data indicates that the system gives accurate temperature information beneath the asphalt layer. To be precise this measurement field was not yet fully covered with the asphalt pavement. The impact of partly missing pavement on the results will be found in measurements made next year at the same time. The full asphalting of the measurement field took place in June 2014.

The future works will include an evaluation of different methods for calculating rate of temperature change, analyzing the data of pyranometer and the heat flux plate and comparison of them to the temperature data measured under the pavement. The weather conditions have to be recorded precisely too. The usability of the asphalted areas for heat collection will be analyzed. The measurements will be continued at least for one whole year to see how different layers warm up and cool down.

## Figures and Tables

**Table 1:** Cable position, material, and height of layer (if applicable).

Cable position (m)	Material/element	Height of layer (m)
355–369	Sand	0.10
369–383	Gravel	0.10
383–390	Asphalt	0.05
390–408	Air	0.13
408-409	60 W lamp bulb	—

**Table 2:** Date, time, and duration of asphalt heating experiment.

Date	Time	Duration
15.1.2013	8:16–13:26	5 h 10 min
16.1.2013	8:11–15:13	7 h 2 min
17.1.2013	8:25–15:31	7 h 6 min
18.1.2013	7:50–15:15	7 h 22 min
